# Evaluation of a microfluidic-based point-of-care prototype with customized chip for detection of bacterial clusters

**DOI:** 10.1128/spectrum.00862-24

**Published:** 2024-11-06

**Authors:** Janina Treffon, Nicole Isserstedt-John, Richard Klemm, Claudia Gärtner, Alexander Mellmann

**Affiliations:** 1 University Hospital Münster, Institute of Hygiene, Münster, Germany; 2 microfluidic ChipShop GmbH, Jena, Germany; Columbia University, New York, New York, USA

**Keywords:** point-of-care system, microfluidic chip, cluster detection, single-nucleotide polymorphisms, real-time PCR

## Abstract

Bacterial infection clusters cause high mortality rates and healthcare costs due to excessive therapy and hygiene measures. The aim of this study was to develop an automated real-time PCR-based point-of-care (POC) system with a customized microfluidic chip that facilitates fast detection of bacterial cluster isolates by targeting cluster-specific single-nucleotide polymorphisms (SNPs). For cluster detection of *Acinetobacter baumannii*, *Staphylococcus aureus*, and *Escherichia coli*, nine TaqMan real-time PCR assays targeting cluster-specific SNPs were designed. Additionally, for DNA input control, a universal PCR amplifying the 16S rDNA was constructed. All reactions were implemented into a microfluidic chip that was analyzed by a POC prototype enabling automated sample processing, fluid handling, and signal detection. Performance of the prototype was evaluated using 45 chips loaded with defined bacterial solutions, including swab eluates. For seven PCRs, implementation into the microfluidic chip was successful, leading to correct identification of all SNPs specific for *A. baumannii* and *E. coli* cluster isolates and delineation of all non-cluster strains within 70 min. The remaining three reactions failed in the chip, which resulted in misidentification of the *S. aureus* cluster. Sensitivity, specificity, and accuracy of the prototype were 43%, 88%, and 55%, respectively. The detection limit was PCR dependent and ranged between 10^3^ and 10^5^ colony-forming units/mL. Once optimized, the microfluidic POC system for cluster detection could be applied as time-saving and easy-to-use method to complement whole-genome sequencing efforts during screening for bacterial clusters.

**IMPORTANCE:**

Especially in medical facilities, where morbid people are nursed in close distance to each other, pathogenic bacteria can accumulate and spread. To contain such infection clusters, usually time- and labor-intensive large-scale screening assays are conducted, where patients and patient-side surfaces are sampled, and PCR or whole-genome sequencing analyses are conducted to confirm or deny cluster affiliation of cultivated bacteria. Hence, fast solutions with easy application are required to complement the current state-of-the-art technology for cluster surveillance. Here, we developed a fully automated microfluidic point-of-care prototype that identified bacterial cluster isolates within 70 min from bacterial solutions, including swab eluates. The system requires only low hands-on time and can be applied apart from laboratory infrastructures near the patient, which considerably reduces the time from sampling to result. This ensures fast implementation of hygiene measures and quick containment of the infection cluster, which would enhance patients’ safety and outcome.

## INTRODUCTION

Fast and accurate identification of pathogens is important for successful treatment of infectious diseases as it can facilitate immediate initiation of an appropriate therapy and enhances patients’ outcomes (1). While classical protractive culture-based methods are still the gold standard for pathogen identification, nowadays, additionally, point-of-care (POC) tests are used to detect infectious viruses, bacteria, and parasites and their resistances. In contrast to culture-based methods, these tests are applied in close proximity to the patient and deliver results within minutes to hours so that therapy or hygiene measures can be implemented while the patient is still in the medical facility (2, 3). Many diagnostic POC tests are based on nucleic acid amplification technologies such as PCR and allow for the detection of specific genes in patient samples starting from DNA or RNA with high sensitivity (4, 5). For near-patient testing apart from laboratories, these assays can be integrated into fully automated microfluidic systems (6–9). As these systems combine automated sample processing, fluid handling, and signal detection, they require only low hands-on time and can easily be implemented into the daily hospital routine. This reduces costs for laboratory consumables and personnel, decreases the time from sampling to detection (3), and increases consistency and safety during handling of contagious samples.

Especially during outbreak events, fast, easy, and accurate pathogen detection is crucial. Bacterial outbreak clusters are accumulations of genetically identical or highly similar bacterial strains with epidemiological context (10, 11). In particular, clusters comprising antibiotic-resistant strains provoke high mortality rates and enormous healthcare costs due to excessive medications and hygiene measures (12, 13). To contain the cluster and monitor infection control measures, large-scale screening assays are conducted, where patients and patient-side surfaces are sampled for the presence of bacteria that belong to the cluster (14). These so-called cluster isolates have to be differentiated from sporadic bacterial strains that do not belong to the cluster, i.e., non-cluster isolates.

In a previous study, we developed a cluster typing pipeline within 7 days that enabled differentiation between cluster isolates and sporadic strains via cluster-specific single-nucleotide polymorphisms (SNPs) using conventional real-time PCR methods (15). Here, we augmented the system and developed a prototype of a fully automated real-time PCR-based POC system with a customized microfluidic chip that facilitates detection of bacterial clusters in nearly 1 h directly from swab samples. System functionality was tested retrospectively on three outbreak clusters of the clinically relevant pathogens *Acinetobacter baumannii*, *Escherichia coli*, and *Staphylococcus aureus*. We expect that the POC system can be established within 14 days after outbreak detection. Subsequently, it can be applied in addition to conventional typing methods to improve outbreak surveillance.

## MATERIALS AND METHODS

### Bacterial strains and cultivation

In total, 42 bacterial clinical and type strains of the following species were used to validate real-time PCR: *Acinetobacter baumannii*, *Acinetobacter calcoaceticus, Acinetobacter dijkshoorniae, Acinetobacter nosocomialis*, *Acinetobacter pittii*, *Acinetobacter seifertii, Enterobacter cloacae, Enterococcus faecium, Escherichia coli, Klebsiella pneumoniae*, *Pseudomonas aeruginosa, Staphylococcus aureus,* and *Staphylococcus epidermidis* (Table S1). While type strains were named according to their official culture collection number, clinical isolates were labeled with an internal number (prefix “OC”). Sixteen strains belonged to three bacterial clusters that were identified at the University Hospital Münster, Germany (Fig. S1). The first cluster comprised three *A. baumannii* strains that were collected during routine screening in July 2008. The second cluster consisted of six *E. coli* strains, which were received from January until February 2015 (16). The third cluster contained seven methicillin-resistant *S. aureus* strains that were sampled between October 2016 and November 2017 at the obstetrics and neonatal departments (17).

Prior to extraction of genomic DNA for real-time PCR analysis or whole-genome sequencing (WGS), bacteria were cultured on Columbia sheep blood agar (Oxoid Deutschland GmbH, Wesel, Germany) at species-specific temperatures (30°C–37°C). For tests in microfluidic chips, bacteria were cultivated overnight in lysogeny broth medium at species-specific temperatures (30°C–37°C) and 180 rpm. Overnight cultures were centrifuged for 3 min at 14,000 rpm, and bacterial pellets were washed two times with 0.9% NaCl solution. For chip analysis, NaCl solutions were inoculated with either one single bacterial strain or up to six different bacterial strains at the indicated concentration. More details about bacterial strains analyzed here are given in the supplemental material.

### Construction of WGS reference data sets

To confine the number of cluster-unspecific SNPs during SNP search, for each analysis a species-specific WGS reference data set was constructed using the software SeqSphere^+^ (18). The *A. baumannii* WGS reference data set comprising 481 *A*. *baumannii* genomes was built as described in Treffon et al. (15). The *S. aureus* and the *E. coli* WGS reference data sets were constructed in the same manner. Details are given in the supplemental material. In brief, all *S. aureus* and *E. coli* complete genomes and chromosomes that were freely available on 22 July 2022 and 31 August 2022, respectively, were downloaded from NCBI Genome database (https://www.ncbi.nlm.nih.gov/genome/). Species level was confirmed by Mash Screen analysis (19), and genomes were analyzed with either *S. aureus* or *E. coli* core genome multilocus sequence typing (cgMLST) scheme, respectively (20, 21). In both data sets, genomes exhibiting <95% of cgMLST genes were excluded. Based on the cgMLST profiles, for each data set, a minimum spanning tree (MST) was generated to group similar genotypes. In these MSTs, *S. aureus* and *E. coli* genomes with an allelic distance of ≤24 and ≤10, respectively, belonged to the same cluster. Both WGS data sets were scaled down to 450 *S*. *aureus* and 472 *E. coli* heterogeneous, non-clustering genomes. All *S. aureus* and *E. coli* genomes used to construct the WGS reference data sets are listed in Table S2.

### Detection of cluster-specific SNPs and alignment analysis with NCBI Nucleotide BLAST

Identification of specific SNPs for the *A. baumannii*, the *E. coli*, and the *S. aureus* cluster was done with the software tool SeqSphere^+^ version 9.0 (18) running on a server with Intel Xeon Gold 6254 CPU @ 3.10 GHz (two processors) and 256 GB of RAM under control of the operating system Microsoft Windows Server 2019 Standard. Using a computer with less CPU and RAM is possible but will extend analysis time. To verify the cluster specificity of the SNPs, the SNPs and their neighboring nucleotides were screened for their absence in other *Acinetobacter*, *E. coli*, and *S. aureus* genomes, respectively, via alignment analysis with the NCBI Standard Nucleotide BLAST web application (https://blast.ncbi.nlm.nih.gov/Blast.cgi?LINK_LOC=blasthome&PAGE_TYPE=BlastSearch&PROGRAM=blastn) (22, 23). Analysis was performed as described recently (15). Details are listed in the supplemental material.

### Extraction of genomic DNA

Genomic DNA analyzed by real-time PCR or WGS was extracted as described by Treffon et al. (15). Additional information is given in the supplemental material.

### Whole-genome sequencing

Bacterial strains *E. coli* OC293, OC306, OC307, OC312 and *S. aureus* OC261, OC262, OC273, OC278 were subjected to WGS on a MiSeq platform (Illumina Inc., San Diego, CA, USA). WGS and subsequent *de novo* assembly was performed as recently described (24).

### Design of probe-based real-time PCR

To detect cluster isolates specifically, TaqMan real-time PCRs were established. Each reaction contained two probes: one probe that binds to cluster-specific SNPs in DNA of cluster isolates and another probe that targets the alternative nucleotide at SNP position in DNA of non-cluster strains. For primer and probe design, cluster-specific SNPs and their surrounding nucleotides were subjected to OligoArchitect Online (http://www.oligoarchitect.com/LoginServlet) provided by Sigma-Aldrich. The function “Dual-Labeled Probe” was run with default settings, and only oligonucleotide concentrations were adapted. For primers and probes targeting the *E. coli* cluster, the concentrations were changed to 400 and 200 nM, respectively. As preliminary laboratory tests revealed that PCR functionality was enhanced with primer and probe concentrations of 200 and 400 nM, respectively, during PCR design for *A. baumannii* and *S. aureus* clusters, the tool settings were accordingly adapted.

Per cluster, three cluster-specific SNPs with good or best quality (if available) according to OligoArchitect were subjected to synthesis by Sigma Aldrich (Table S3): Seq1AB, Seq7AB, and Seq12AB for detection of *A. baumannii* cluster isolates; Seq15EC, Seq25EC, and Seq31EC to confirm presence of *E. coli* cluster isolates; and Seq1SA, Seq4SA, and Seq5SA for identification of *S. aureus* cluster isolates. To control DNA input, the same 16S rDNA-specific PCR as described in our previous study was used (15) (Table S3).

### Implementation of probe-based real-time PCR

**Fig 1:**
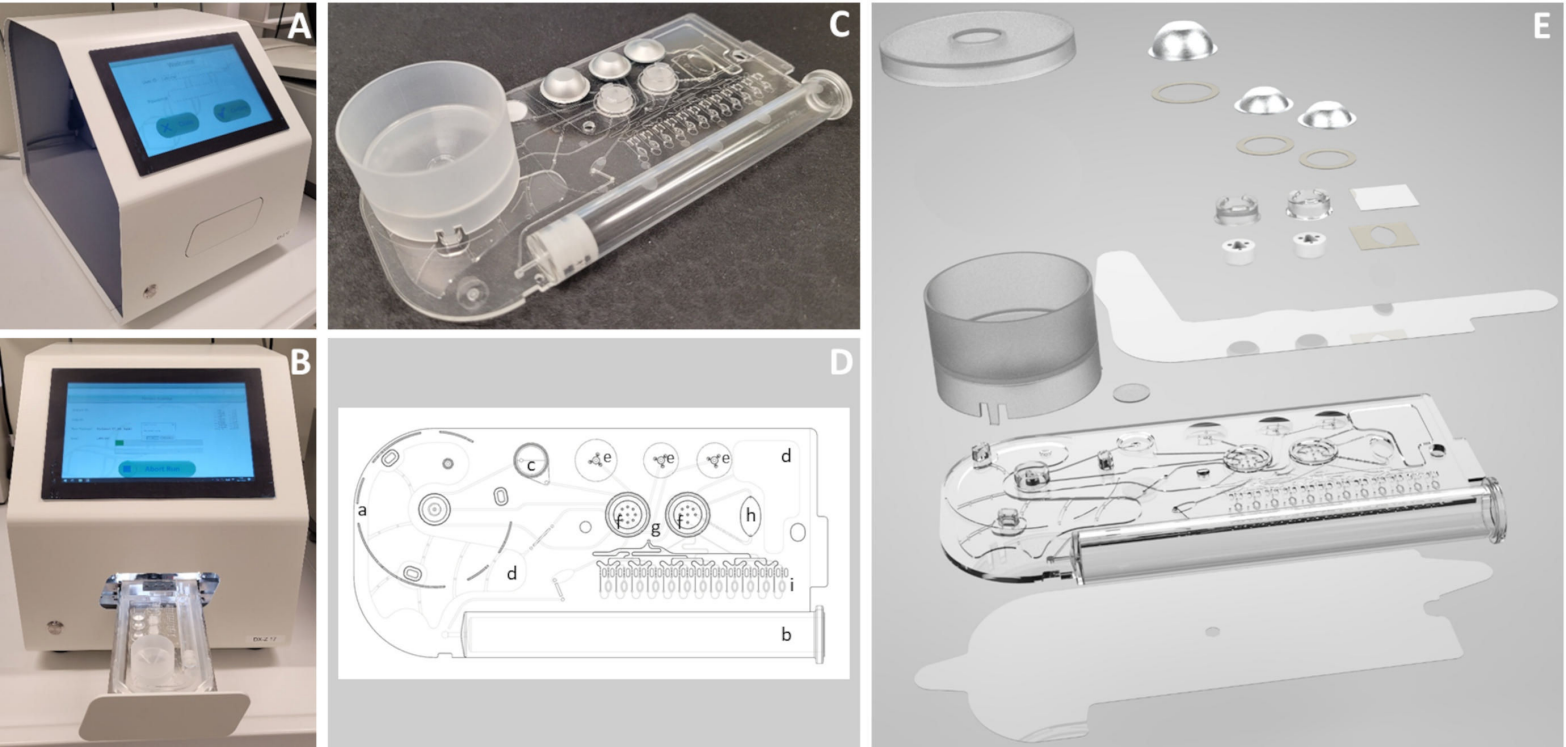
ChipGenie edition Dx and customized microfluidic chips. (A) ChipGenie edition Dx (25) enabling sample preparation and real-time PCR analysis. (B) ( ChipGenie edition Dx with microfluidic chip. (C–E) Photo, 2D scheme, and exploded view of microfluidic chip with sample tank (a), syringe pump (b), filter (c), wastes (d), blisters containing buffer for sample washing, water for DNA elution, and the Biozym probe qPCR mix (Biozym Scientific GmbH, Hessisch Oldendorf, Germany) (e), turning valves (f), metering channel (g), mix chamber (h), and 12 PCR cavities (i). 2D scheme and exploded-view drawing were provided by microfluidic ChipShop (Jena, Germany) and were slightly modified. The sample is added to the sample tank, and the chip is inserted into the analyzer. By movement of the syringe pump, the sample is soaked into the chip and flows over a filter, where bacterial cells are collected. The cells are washed with buffer stored in the first blister. Subsequently, the bacterial cells are lysed by 12 cycles of sonication to release DNA. The DNA is eluted from the filter by water stored in the second blister. A small volume of the sample is stored in the metering channel located between both rotary valves. In the next step, this volume is diluted with the Biozym probe qPCR mix released from the last blister. The diluted sample flows into the mix chamber, and all components are mixed via sonication. Finally, the sample containing bacterial DNA and all master mix components is distributed to the PCR chambers that contain primers and probes, and real-time PCR is performed. During the whole process, liquids are moved via turning valves and syringe pump. Flow-through is collected in the waste containers.

Prior to implementation of primers and probes in the microfluidic chip, functionality of real-time PCRs was verified in white, high-profile 96-well plates (Bio-Rad Laboratories GmbH, Feldkirchen, Germany) loaded with 10 µL reactions. The plates were analyzed on a CFX96 Touch Real-Time PCR Cycler (Bio-Rad Laboratories GmbH) running with the following thermal cycling conditions: 95°C for 2 min, 40 cycles including 95°C for 15 s and 64°C for 1 min (signal detection), followed by 20°C for 3 min. Lid temperature and ramp rate were set to 100°C and 5.0°C/s, respectively. Evaluation of real-time PCRs was done with Bio-Rad CFX Maestro 1.1 Software (Bio-Rad Laboratories GmbH), applying Cq determination mode regression to cycles 5–40. Afterward, PCRs were implemented in customized microfluidic chips combining sample preparation and real-time PCR with 12 × 5 µL reaction cavities. Chips were analyzed on a ChipGenie edition Dx (25) provided by microfluidic ChipShop GmbH (Jena, Germany) (Fig. 1). To reduce analysis time, for real-time PCR in microfluidic chips, a shortened cycler protocol was applied, which was also validated in well plates: 95°C for 3 min followed by 40 cycles including 95°C for 10 s and 64°C for 20 s (signal detection). Evaluation of real-time PCRs in microfluidic chips was done with TSO Analyzer software 2.0.0.19 (microfluidic ChipShop GmbH). As soon as PCR was finished, the TSO analyzer software generated a report that contained information regarding Ct values and target presence or absence (Fig. S2). Ct values are calculated with a dynamic baseline and inflection point identification. A threshold value is only used as an exclusion criterion once the amplification is below meaningful endpoint levels.

Irrespective of the PCR setting (i.e., well plate or microfluidic chip), each SNP-specific reaction contained 200 nM of forward and reverse primers, 400 nM of SNP-specific and alternative probe, 1× Biozym Probe qPCR Mix (Biozym Scientific GmbH, Hessisch Oldendorf, Germany), and bacterial DNA. The 16S rDNA-specific reaction contained 900 nM of forward and reverse primers, 400 nM of 16S rDNA-specific probe, 1× Biozym probe qPCR mix (Biozym Scientific GmbH), and bacterial DNA.

Functionality of cluster-specific primers and probes in 96-well plates was evaluated by monitoring real-time amplification of 100 and 10,000 copies of DNA (0.375 and 37.5 pg, respectively) extracted from cluster isolates and 10,000 copies of DNA isolated from non-cluster isolates, assuming that on average one copy of bacterial DNA corresponds to 3.75 fg. For tests in microfluidic chips, solutions with cluster isolates were set to 10^5^ colony-forming units (CFU)/mL, while non-cluster isolates were analyzed with 10^7^ CFU/mL. We applied lower amounts of (DNA extracted from) cluster strains than of (DNA extracted from) non-cluster strains to demonstrate PCR functionality even in a worst case scenario, where huge amounts of non-cluster isolates are present, but only low numbers of cluster strains are available. To determine the detection limit in the microfluidic chip, additional concentrations of 10^4^, 10^3^, and 10^2^ CFU/mL were applied. Generally, 3 mL of each solution was filled into the sample tank of the microfluidic chip, the chip was inserted into the analyzer, and the microfluidic protocol was started. For swab tests, dry swabs were soaked with 0.9% NaCl solution for 20 s and rubbed over the oral mucosa of six healthy volunteers. Afterward, some of the swabs were loaded with 3 µL of a 10^8^ CFU/mL concentrated bacterial solution. All swabs were eluted in 3 mL of NaCl solution, which was analyzed with the microfluidic chip. Before, 10 µL of each eluate was plated on blood agar and incubated overnight at 37°C to control bacterial growth (Fig. S3).

### Statistics

Sensitivity, specificity, and accuracy of the microfluidic detection system were calculated in comparison to WGS data based on the number of true-positive (TP), false-positive (FP), true-negative (TN), and false-negative (FN) results as described by Baratloo et al. (26).

## RESULTS

### Identification and validation of cluster-specific SNPs

Within 8–32 min, SeqSphere^+^ identified 194, 33, and 45 SNPs that were present in the *A. baumannii*, *E. coli*, and *S. aureus* cluster isolates, respectively, but absent in the non-cluster strains of the respective WGS reference data sets (Table 1). Twenty, 33, and 20 SNPs and their surrounding nucleotides were aligned to public *Acinetobacter*, *E. coli*, and *S. aureus* genomes stored in the NCBI Nucleotide Collection database via BLAST analysis to verify the cluster specificity of the SNPs. None of the 20 SNPs identified for the *A. baumannii* and the *S. aureus* cluster could be detected in any of the NCBI genomes, demonstrating their cluster specificity. In contrast, 24 of 33 SNPs detected for the *E. coli* cluster were present in at least one of the public *E. coli* genomes, resulting in only nine SNPs that were unique to the cluster.

**TABLE 1:** Identification of specific SNPs for the *A. baumannii*, the *E. coli*, and the *S. aureus* cluster^*a*^^,^^*b*^

Cluster	Analysis time (minutes)	No. of SNPs identified	BLAST analysis results
AB	32	194 (+ 9 indels)	20 of 20 SNPs not present in other *Acinetobacter* genomes
EC	8	33 (+ 1 indel)	9 of 33 SNPs not present in other *E. coli* genomes
SA	23	45	20 of 20 SNPs not present in other *S. aureus* genomes

^*a*^ Insertions/deletions (indels) were excluded from further analysis.

^*b*^ AB, *A. baumannii* cluster; EC, *E. coli* cluster; SA, *S. aureus* cluster.

### Real-time PCR detection of cluster isolates in 96-well plates

Per cluster, three SNPs that allowed construction of primer and probes with good quality according to OligoArchitect Online tool were selected for design of cluster-specific TaqMan real-time PCR assays: Seq1AB, Seq7AB, Seq12AB (*A. baumannii* cluster), Seq15EC, Seq25EC, Seq31EC (*E. coli* cluster), Seq1SA, Seq4SA, Seq5SA (*S. aureus* cluster). All nine SNPs were located in the core genome of the respective species (Table S4).

**Fig 2:**
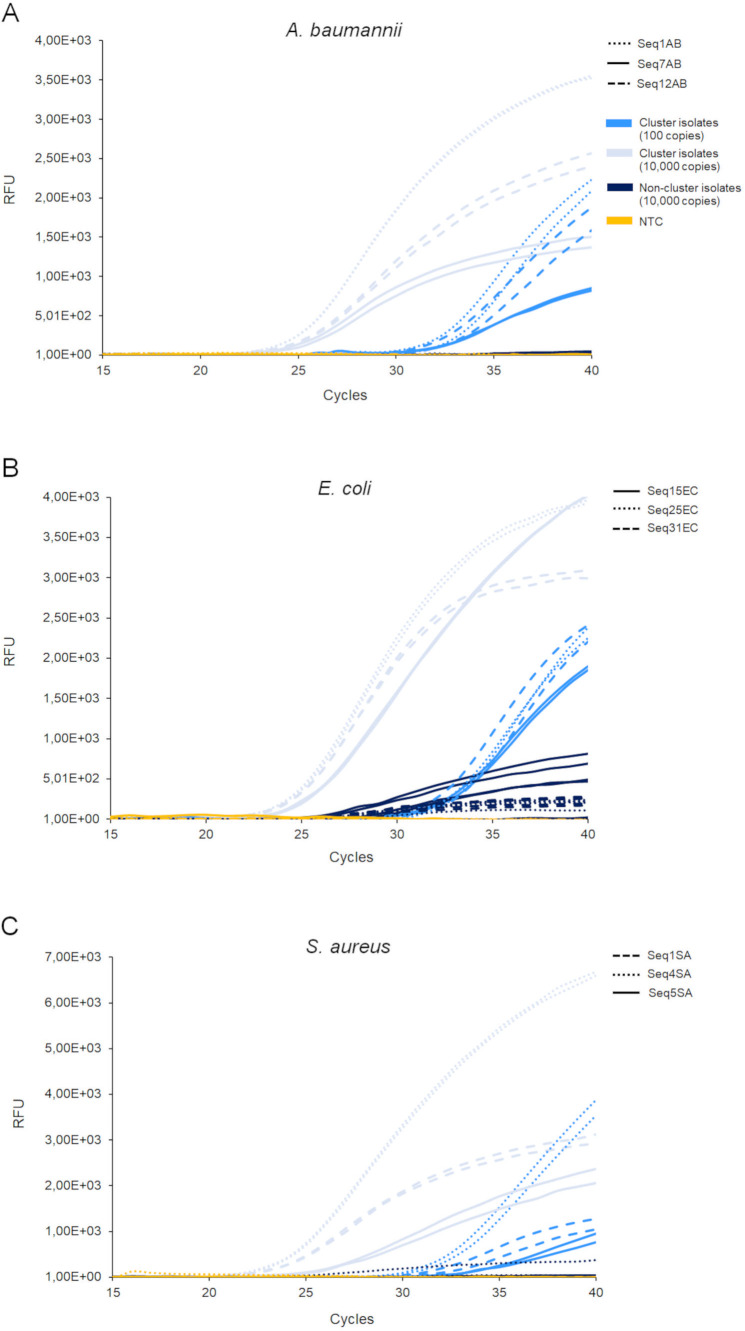
Probe-based real-time PCR detection of cluster isolates in 96-well plates. *A. baumannii* (A), *E. coli* (B), and *S. aureus* (C) cluster isolates were detected via TaqMan real-time PCR assays targeting cluster-specific SNPs. The PCRs were tested on DNA extracted from two cluster isolates (*A. baumannii* OC050 and OC052, *E. coli* OC286 and OC287, *S. aureus* OC248 and OC249) and five non-cluster strains, including the type strain of each species (*A. baumannii* OC045, OC046, OC047, OC049, and DSM 30007, *E. coli* OC293, OC306, OC307, OC312, and DSM 30083, *S. aureus* OC261, OC262, OC273, OC278, and DSM 20231). While DNA of cluster isolates was applied with 100 copies (light blue) and 10,000 copies (middle blue), DNA of non-cluster strains was tested only with 10,000 copies (dark blue). As non-target control (NTC), water instead of DNA was used (yellow). RFU, relative fluorescence units.

Prior to implementation of primers and probes in the microfluidic chip, functionality of real-time PCRs was verified in 96-well plates. Functionality tests with DNA extracted from two cluster isolates and five non-cluster strains per species revealed that all PCRs reliably detected 100 and 10,000 copies of cluster isolate DNA (Fig. 2; Table S5). Five SNP-PCRs (Seq1AB, Seq7AB, Seq12AB, Seq1SA, Seq5SA) were highly cluster specific and did not generate any fluorescence signals with 10,000 copies of DNA extracted from non-cluster isolates. In contrast, the remaining four PCRs (Seq15EC, Seq25EC, Seq31EC, Seq4SA) amplified DNA isolated from at least one non-cluster strain. However, PCR curves of these reactions were considerably flatter than those of cluster-specific reactions (Fig. 2) and still allowed for differentiation of cluster and non-cluster strains.

The 16S rDNA reaction to control DNA input successfully amplified DNA of all three cluster species (Table 2). Admittedly, also the non-target control generated a Ct value, which, however, was higher than the Ct value of reactions containing bacterial DNA.

**TABLE 2:** Probe-based real-time PCR detection of 16S rDNA in 96-well plate^*a*^

Cluster	Species	Strain	DNA copies	Ct value
AB	*A. baumannii*	OC050	100	30.24
AB	*A. baumannii*	OC052	100	29.20
AB	*A. baumannii*	OC050	10,000	21.63
AB	*A. baumannii*	OC052	10,000	21.64
–	*A. baumannii*	OC045	10,000	21.46
–	*A. baumannii*	OC046	10,000	21.55
–	*A. baumannii*	OC047	10,000	21.91
–	*A. baumannii*	OC049	10,000	20.73
–	*A. baumannii*	DSM 30007	10,000	20.84
EC	*E. coli*	OC286	100	30.69
EC	*E. coli*	OC287	100	30.21
EC	*E. coli*	OC286	10,000	21.86
EC	*E. coli*	OC287	10,000	21.36
–	*E. coli*	OC293	10,000	22.41
–	*E. coli*	OC306	10,000	21.79
–	*E. coli*	OC307	10,000	21.31
–	*E. coli*	OC312	10,000	21.67
–	*E. coli*	DSM 30083	10,000	23.58
SA	*S. aureus*	OC248	100	29.20
SA	*S. aureus*	OC249	100	29.25
SA	*S. aureus*	OC248	10,000	20.48
SA	*S. aureus*	OC249	10,000	20.37
–	*S. aureus*	OC261	10,000	19.60
–	*S. aureus*	OC262	10,000	19.31
–	*S. aureus*	OC273	10,000	19.35
–	*S. aureus*	OC278	10,000	19.31
–	*S. aureus*	DSM 20231	10,000	20.97
–	–	H_2_O	–	36.57

^*a*^ AB, *A. baumannii* cluster; EC, *E. coli* cluster; H_2_O, non-target control; SA, *S. aureus* cluster; –, non-cluster strain.

### Real-time PCR detection of cluster isolates in customized microfluidic chips

After successful reaction validation in 96-well plates, SNP- and 16S rDNA-specific PCRs were implemented in the microfluidic chip, and functionality of the whole prototype combining microfluidic chip and analyzer (Fig. 1) was tested. On the microfluidic chip, each SNP-specific reaction was spotted in one PCR cavity, while the PCR for detection of 16S rDNA was spotted in three PCR cavities. In total, 45 chips were produced, which were tested on various bacterial solutions. The runs of seven chips had to be aborted either due to software failures or microfluidic problems. All remaining 38 chips worked well (Table 3). Of these, 29 chips were tested with solutions containing either one single cluster isolate or up to six non-cluster strains to verify chip functionality.

**TABLE 3:** Probe-based real-time PCR detection of cluster isolates in microfluidic chips^*a*^^,^^*b*^^,*c*^

Chip no.	Test	Species	Strain	Cluster	Concen-tration (CFU/mL)	PCR chamber-primer/probe mix											
						1-16S	2-Seq1AB	3-Seq7AB	4-Seq12AB	5-16S	6-Seq1SA	7-Seq4SA	8-Seq5SA	9-Seq15EC	10-Seq25EC	11-Seq31EC	12-16S
1	Functionality	*A. baumannii*	OC050	AB	10^5^	+	+	+	+	+	−	−	+	+	−	+	−
2	Functionality	*A. baumannii*	OC050 (R)	AB	10^5^	+	−	+	+	+	−	−	+	−	−	−	+
3	Functionality	*A. baumannii*	OC051	AB	10^5^	+	+	+	+	+	−	+	−	+	−	−	+
4	Functionality	*A. baumannii*	OC051 (R)	AB	10^5^	+	+	−	−	−	−	−	−	−	−	−	+
5	Functionality	*A. baumannii*	OC052	AB	10^5^	+	+	−	+	+	−	−	−	+	−	−	+
6	Functionality	*S. aureus*	OC248	SA	10^5^	+	−	−	−	+	−	+	−	−	−	−	+
7	Functionality	*S. aureus*	OC249	SA	10^5^	−	+	−	−	+	−	+	−	−	−	−	+
8	Functionality	*S. aureus*	OC249 (R)	SA	10^5^	+	−	−	−	+	−	−	−	−	−	−	+
9	Functionality	*S. aureus*	OC250	SA	10^5^	+	−	+	−	+	−	+	−	−	−	+	+
10	Functionality	*S. aureus*	OC251	SA	10^5^	+	−	−	−	−	−	+	−	−	−	−	+
11	Functionality	*S. aureus*	OC252	SA	10^5^	+	−	−	−	+	−	−	−	−	−	−	+
12	Functionality	*S. aureus*	OC252 (R)	SA	10^5^	+	−	−	−	+	−	−	−	−	−	−	+
13	Functionality	*S. aureus*	OC253	SA	10^5^	+	−	−	−	+	−	−	−	−	−	−	+
14	Functionality	*S. aureus*	OC253 (R)	SA	10^5^	+	−	−	−	+	−	+	−	−	−	−	+
15	Functionality	*S. aureus*	OC254	SA	10^5^	+	−	−	−	+	−	+	−	−	−	−	+
16	Functionality	*E. coli*	OC286	EC	10^5^	+	−	−	−	−	−	−	+	−	+	−	+
17	Functionality	*E. coli*	OC286 (R)	EC	10^5^	+	−	−	−	−	−	−	−	−	+	+	+
18	Functionality	*E. coli*	OC287	EC	10^5^	+	−	−	−	+	−	−	−	−	+	+	+
19	Functionality	*E. coli*	OC288	EC	10^5^	+	−	−	−	+	−	−	−	+	+	+	+
20	Functionality	*E. coli*	OC289	EC	10^5^	+	−	−	−	−	−	−	−	−	+	+	+
21	Functionality	*E. coli*	OC290	EC	10^5^	+	+	−	−	+	−	−	−	−	+	+	+
22	Functionality	*E. coli*	OC291	EC	10^5^	+	−	−	−	−	−	−	−	+	+	+	+
23	Functionality	*A. baumannii*	OC049	nc strain	10^7^	+	−	−	−	+	−	−	−	−	−	−	+
		*S. aureus*	OC261														
		*E. coli*	OC307														
24	Functionality	*A. baumannii*	OC047	nc strain	10^7^	+	−	−	+	+	−	−	−	−	−	−	+
		*S. aureus*	OC273														
		*E. coli*	OC293														
25	Functionality	*A. baumannii*	OC046	nc strain	10^7^	+	+	−	−	+	−	−	−	−	−	−	+
		*S. aureus*	OC262														
		*E. coli*	OC306														
26	Functionality	*A. baumannii*	OC048	nc strain	10^7^	+	−	−	−	+	−	−	−	+	−	−	+
		*S. aureus*	OC278														
		*E. coli*	OC312														
27	Functionality	*E. cloacae*	DSM 30054	nc strain	10^7^	+	−	−	−	+	−	−	−	+	−	−	+
		*E. faecium*	DSM 20477														
		*E. coli*	DSM 30083														
28	Functionality	*K. pneumoniae*	DSM 30104	nc strain	10^7^	+	−	−	−	−	−	−	−	−	−	+	+
		*P. aeruginosa*	DSM 50071														
		*S. aureus*	DSM 20231														
		*S. epidermidis*	DSM 20044														
29	Functionality	*A. baumannii*	DSM 30007	nc strain	10^7^	+	−	−	−	+	−	−	−	−	−	−	+
		*A. seifertii*	DSM 102854														
		*A. nosocomialis*	DSM 102856														
		*A. pittii*	DSM 25618														
		*A. calcoaceticus*	DSM 30006														
		*A. dijkshoorniae*	LMG 29605														
30	Swab sample	*A. baumannii*	OC050	AB	10^5^	+	+	+	+	+	−	−	−	+	−	−	+
31	Swab sample	*E. coli*	OC286	EC	10^5^	+	−	−	−	+	−	−	−	−	+	+	+
32	Swab sample	–	–	nc strain	–	+	−	−	−	+	−	−	−	−	−	−	+
33	Detection limit	*A. baumannii*	OC050	AB, SA, and EC	10^5^	+	+	+	+	+	−	+	−	−	+	+	+
		*S. aureus*	OC248														
		*E. coli*	OC286														
34	Detection limit	*A. baumannii*	OC050	AB, SA, and EC	10^4^	+	+	−	−	+	−	−	−	−	+	+	+
		*S. aureus*	OC248														
		*E. coli*	OC286														
35	Detection limit	*A. baumannii*	OC050	AB, SA, and EC	10^3^	+	+	−	−	−	−	−	+	+	+	+	+
		*S. aureus*	OC248														
		*E. coli*	OC286														
36	Detection limit	*A. baumannii*	OC050	AB, SA, and EC	10^2^	+	−	−	−	−	−	−	−	−	−	−	+
		*S. aureus*	OC248														
		*E. coli*	OC286														
37	Detection limit	NaCl	–	nc strain	–	+	+	−	−	−	−	−	−	−	−	−	+
38	Detection limit	NaCl	- (R)	nc strain	–	+	−	−	−	−	−	−	−	−	−	−	+

^*a*^ Results of seven chips that failed due to software or microfluidic problems are not shown.

^*b*^ AB, *A. baumannii* cluster; EC, *E. coli* cluster; R, repeated experiment; SA, *S. aureus* cluster; nc strain, non-cluster strain; +, positive PCR according to software; −, negative PCR according to software.

^*c*^ Dark grey, false positive result; light grey, False negative results.

All 29 chips were valid, which means that at least two out of three 16S rDNA PCRs were positive, indicating that bacterial DNA could be extracted from the sample. In detail, 20 chips had 3 positive 16S rDNA signals. Nine chips showed only two positive 16S rDNA reactions. Of these, in seven chips, the 16S rDNA reaction spotted in PCR cavity 5 did not yield any signal.

Sixteen of 29 chips identified cluster and non-cluster strains correctly, which means that at least two of three or maximally one of three cluster-specific SNP reactions were positive, when either a cluster or a non-cluster isolate was present, respectively. In detail, nine chips properly identified the respective *A. baumannii* or *E. coli* cluster isolate that was added to the sample solution. Strikingly, the PCR targeting Seq15EC often failed and gave either false-positive or false-negative signals. Seven chips correctly indicated the presence of non-cluster isolates as none or only one SNP was detected. The remaining 13 chips showed unexpected results. Among 12 chips, 10 chips are used for the detection of the *S. aureus* cluster, indicating the absence of a cluster isolate, although a cluster isolate was added to the sample solution. Detection of *S. aureus* cluster isolates failed due to Seq1SA- and Seq5SA-specific PCRs, which did not deliver any positive signal at all. The 13th chip indicated the presence of *A. baumannii* and *E. coli* cluster isolates, although only an *A. baumannii* cluster isolate was added to the sample solution.

The 29 chips were selected to calculate sensitivity, specificity, and accuracy of the cluster detection system in comparison to WGS data as described elsewhere (26). We defined that a positive chip indicated the presence of a cluster isolate, while a negative chip was indicative for the absence of a cluster isolate but the presence of a non-cluster strain in the sample solution. Of all 29 chips, 10 chips were positive, and 19 chips were negative (Table 4). Nine chips were true positive (cluster isolate present and detected), and one chip (no. 1, Table 3) was false positive (*E. coli* cluster isolate absent, but detected). Seven chips were true negative (cluster isolate absent and not detected), and 12 were false negative (cluster isolate present, but not detected), among them all chips used for the detection of *S. aureus* cluster isolates. Accordingly, sensitivity, specificity, and accuracy of the microfluidic prototype were 43%, 88%, and 55%, respectively.

**TABLE 4:** Statistical analysis of the microfluidic detection system^*a*^

	Cluster isolate present	Cluster isolate absent, non-cluster isolate present	
Chip positive	9 (TP)	1 (FP)	Accuracy = 55%
Chip negative	12 (FN)	7 (TN)	
	Sensitivity = 43%	Specificity = 88%	

^*a*^ FN = false negative, FP = false positive, TN = true negative, TP = true positive.

To test the system applicability to patient samples, six chips were used for the analysis of eluates from swabs that were rubbed over the oral mucosa of healthy volunteers. Some of the swabs were spiked with cluster isolates. Three of six swab eluates (two containing a cluster isolate and one containing only the bacterial flora of the oral mucosa) were correctly analyzed with the microfluidic system (no. 30–32, Table 3; Fig. S3). However, the analysis of the remaining three eluates failed due to microfluidic problems.

In six chips (no. 33–38, Table 3), solutions containing 10^5^, 10^4^, 10^3^, 10^2^, and 0 CFU/mL of cluster strains *A. baumannii* OC050, *E. coli* OC286, and *S. aureus* OC248 were added to investigate the detection limit of the reactions. While the reactions targeting 16S rDNA, Seq1AB, Seq25EC, and Seq31EC reliably detected down to 10^3^ CFU/mL of cluster strains, reactions targeting Seq7AB, Seq12AB, and Seq4SA detected not less than 10^5^ CFU/mL of cluster isolates (Table 3). In contrast, the PCRs specific for Seq1SA, Seq4SA, and Seq15EC failed in all dilutions. Unfortunately, two of three 16S rDNA-specific reactions gave positive signals in an NaCl solution without bacteria.

## DISCUSSION

The automation of manual laboratory processes reduces costs for consumables and personnel and enables consistent and safe sample handling and analysis within a short time (2, 3). Especially in diagnosis of infectious diseases, fast and accurate examination of contagious patient samples is important to rapidly implement suitable treatment strategies and to prevent contamination by the pathogenic agent. In our study, we developed and evaluated a fully automated microfluidic POC prototype that identified bacterial cluster isolates via detection of cluster-specific SNPs within nearly 1 h from bacterial solutions, including swab eluates. To our knowledge, this is the first POC system that is applicable for bacterial cluster surveillance. As the microfluidic chip is a flexible platform that enables implementation of user-defined PCRs during manufacturing process, fast assay adaptation to new emerging pathogens is supported.

**Fig 3:**
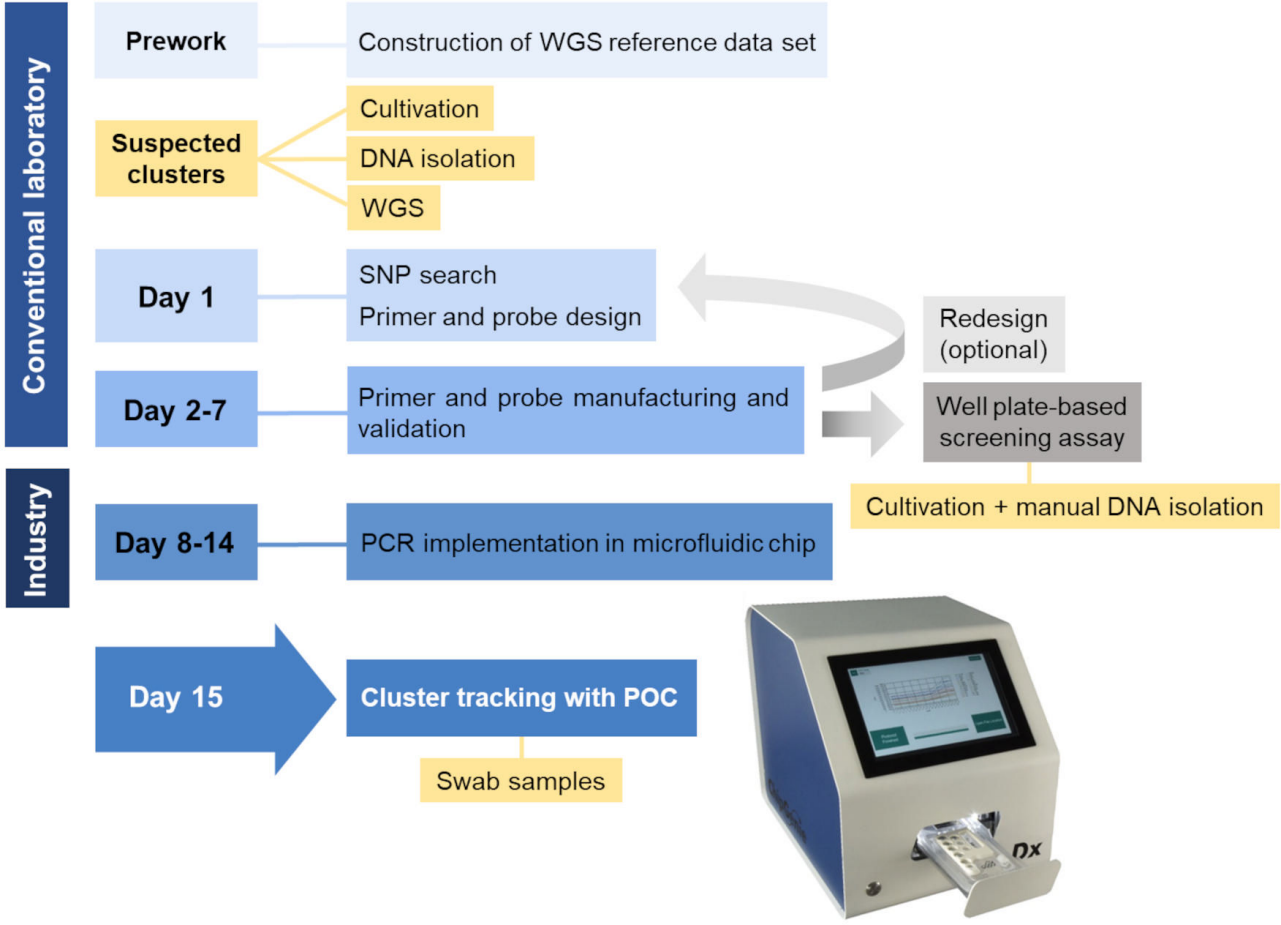
Workflow and turnaround time of cluster screening with well plate-based screening assay or microfluidic POC prototype. The figure was adapted from Treffon et al. (15). Redesign is only required, if PCRs fail during validation. To minimize the risk of redesign, backup PCRs should be designed and manufactured that can be used alternatively in case some PCRs fail. After successful primer and probe manufacturing and validation, a well plate-based screening assay is established that can be already used to track bacterial cluster isolates. However, bacterial cultivation and manual DNA isolation are still required. In contrast, upon PCR implementation in the microfluidic system, swab samples can be analyzed in an automated way, making isolate cultivation and manual DNA isolation unnecessary.

In previous studies, we determined the turnaround time to establish PCR-based screening assays for bacterial clusters performed in 96-well plates, which ranged from 4 to 13 days (15, 27). Although PCR implementation in the microfluidic chip could extend the time for system development for additional 7 days (Fig. 3), screening for cluster isolates with the microfluidic POC system would be much easier and faster compared to the 96-well plate format as neither cultivation nor manual DNA isolation is required. Of course, prior to the final release, there is still need for system optimization as described in detail below.

To set up the system, cluster-specific SNPs were identified. Per cluster, three SNPs were selected for the design of cluster-specific TaqMan real-time PCR assays. All SNP-specific PCRs—and also the 16S rDNA control PCR—were functional in 96-well plates and specifically detected down to 100 copies of DNA extracted from cluster isolates (Fig. 2; Table 2; Table S5). Once implemented in the microfluidic chip, the detection limit of the reactions increased from 100 DNA copies (corresponding to 10^2^ CFU) to 10^3^ or 10^5^ CFU/mL. This indicates that (i) cell lysis and DNA recovery with our prototype were not as efficient as column-based DNA extraction done manually in the laboratory and (b) real-time PCR in the chip was not as sensitive as in the well plate. However, the loss of sensitivity in the chip prevented the detection of unspecific DNA amplification that happened in four SNP-PCRs (Seq15EC, Seq25EC, Seq31EC, Seq4SA) in the well plate (Fig. 2; Table S5). In addition, the detection limit of the prototype is comparable to other real-time PCR-based POC systems that identify down to 10^3^ viral or bacterial DNA copies per milliliter and that are either in development (7) or already available on the market (https://www.biomerieux.com/corp/en/our-offer/clinical-products/biofire-filmarray-pneumonia-panels.html). Admittedly, there exist POC technologies based on real-time PCR, loop-mediated isothermal amplification, or rolling loop amplification that can detect less than 1,000 or even fewer than 100 bacterial cells or viral particles (9, 28–31).

Unfortunately, two of three 16S rDNA-specific reactions became positive in an NaCl solution lacking bacteria (Table 3), a problem that was already observed in the well plate (Table 2). Reasons for this might be (i) the high concentration of oligonucleotides in the reaction, which could cause elevated levels of background fluorescence, or (ii) a contamination of PCR reagents, such as the DNA polymerase with traces of bacterial DNA originating from its production process (32). To prevent misinterpretation of samples with unknown bacterial concentration, we suggest that (i) the 16S rDNA reaction should be optimized for the reduction of background signals or that (ii) a cutoff for this reaction should be implemented into the software that automatically excludes results, which are above a distinct Ct value.

Chip functionality tests revealed that 29 out of 29 chips were valid (i.e., at least two out of three 16S rDNA-PCRs got positive). Furthermore, 16 out of these 29 chips correctly identified cluster strains (i.e., at least two out of three SNP-PCRs got positive) and non-cluster isolates (i.e., maximally one out of three SNP-PCRs got positive). However, some samples had to be analyzed twice for proper identification. Among the correct identified cluster strains, all three *A. baumannii* and all six *E. coli* cluster isolates were located. In addition, for six of seven *S*. *aureus* cluster isolates, at least the Seq4SA-specific reaction was positive, which demonstrates that the prototype is able to lyse gram-negative and -positive bacteria properly. Nevertheless, as the two remaining *S. aureus*-PCRs (Seq1SA, Seq5SA) failed on each chip, *S. aureus* detection was not successful according to our definition.

The failures during *S. aureus* detection diminished sensitivity and accuracy of the prototype to 43% and 55%, respectively. When the 10 chips used for *S. aureus* cluster detection were excluded from statistics, sensitivity and accuracy raised to 82% and 84%, respectively. The sensitivity and specificity values of our prototype after exclusion of chips used for *S. aureus* detection (82% and 88%, respectively) are within the range or only slightly below the values of other PCR-based POC tests (77%–100% and 93%–100 %, respectively) (8, 9, 33, 34). However, compared to other studies (8, 9), we used only a very low total number of chips for statistical analysis (i.e., 29 with and 19 without *S. aureus* detection), which permits only a first impression of system functionality.

The implementation of backup reactions on the microfluidic chip (i.e., three 16S rDNA- and three SNP-PCR spots) facilitated chip evaluation even in case some PCRs failed. This demonstrates that, in the microfluidic chip, PCRs did not perform as reliable as in the well plate. A reason for this could be the complexity of fully automated microfluidic systems that combine sample preparation, fluid handling, and signal detection in one device. All processes are reduced to the essentials, which allows a fast workflow but also involves certain risks. “Quick and dirty” DNA extraction by sonication and elution without column-based purification could provoke the accumulation of inhibitory substances that might hamper PCR. Furthermore, mechanical or user-based mistakes during sample application could lead to air inclusions that might affect the microfluidic flow and result in incomplete filling of the PCR cavities. In addition, small irregularities in chip form or failures during chip insertion process could influence the contact pressure between chip and heating plate whereby certain PCR cavities might not reach the temperatures they should. If PCR oligonucleotides or enzymes are susceptible to such influences, they might fail. Therefore, ideally primer and probes with e.g., broad temperature profile should be implemented in the chip. Strikingly, all dysfunctional reactions (Seq1SA, Seq5SA, Seq15EC, 16S rDNA-specific PCR spotted in PCR cavity 5) were spotted in PCR cavities located the center of the chip. We therefore assume that especially the chip center is error-prone. Application of another annealing temperature or a different PCR buffer or polymerase might stabilize PCRs and could maintain their functionality even under suboptimal conditions in the chip.

In addition, further optimization should be done regarding the analysis of real patient samples. Although the prototype managed to analyze three swab eluates containing bacteria of the oral mucosa and cluster strains (Table 3; Fig. S3), during the analysis of the remaining eluates the microfluidic chip was presumably clogged by swab material or mucosal cells, leading to microfluidic dysfunction and PCR failure. A microfluidic protocol specifically adapted to the sample matrix in terms of sample input, flow rate, and ventilation or the use of a bacteria-collecting filter with wider pores could help to fix this problem.

Summarized, we developed a prototype of a microfluidic fully automated POC system with customized microfluidic chip that specifically identified gram-positive and gram-negative bacterial cluster isolates via detection of cluster-specific SNPs by probe-based real-time PCR within 70 min from bacterial solutions, including swab eluates. Implementation of several SNP-reactions per cluster assured proper cluster assignment even in case some PCRs failed. The flexible chip design allows quick adaptation to new clusters. The system can be established within 14 days upon detection of a bacterial cluster. Implementation of an optimized version of the system for surveillance screenings in addition to other methods would support fast and specific cluster detection.

## Data Availability

All Illumina raw reads of the genomes sequenced in this study were submitted to the European Nucleotide Archive (http://www.ebi.ac.uk/ena/) under Project ID PRJEB65442. The NCBI accession numbers of all other genomes are listed in Tables S1 and S2. Part of this study was presented at the 5th Munich Point-of-Care Testing Symposium: New Horizons for Cross-Sectional Technologies and Extended Application Areas in 2022 and the 75th Annual Meeting of the German Society for Hygiene and Microbiology e.V. in 2023.

## SUPPLEMENTAL MATERIAL

Supplemental material spectrum.00862-24-s0001.docx Supplemental methods, tables, and figures.

Table S2 spectrum.00862-24-s0002.xlsx Genomes used for construction of the *S. aureus* and *E. coli*.

ASM does not own the copyrights to Supplemental Material that may be linked to, or accessed through, an article. The authors have granted ASM a non-exclusive, world-wide license to publish the Supplemental Material files. Please contact the corresponding author directly for reuse.
