# Supplementary material for: Evaluation of a microfluidic-based point-of-care prototype with customized chip for detection of bacterial clusters
Source: Microbiol Spectr. 2024 Nov 6;12(12):e00862-24. doi: 10.1128/spectrum.00862-24 (PMC11619353; doi:10.1128/spectrum.00862-24)
Supplement: Supplemental material — Supplemental methods, tables, and figures. [file spectrum.00862-24-s0001.docx]

**Evaluation of a microfluidic-based point-of-care prototype with customized chip for detection of bacterial clusters**

Janina Treffon^a^, Nicole Isserstedt-John^b^, Richard Klemm^b^, Claudia Gärtner^b^, Alexander Mellmann^a#^

^a^University Hospital Münster, Institute of Hygiene, Münster, Germany

^b^microfluidic ChipShop GmbH, Jena, Germany

^#^Corresponding author: [Alexander.Mellmann@ukmuenster.de](mailto:Alexander.Mellmann@ukmuenster.de)

# Supplemental material and methods

## Bacterial strains

Sequence types of all *Acinetobacter baumannii*, *Escherichia coli*, and *Staphylococcus aureus* strains were determined via *A. baumannii* multilocus sequence typing (MLST) Pasteur (1) and Oxford (2), *E. coli* MLST Warwick (3), and *S. aureus* MLST (4), respectively, using SeqSphere^+^ (5) (Table S1). With the same software, similarity of genotypes was visualized by constructing a minimum spanning tree (MST) based on the allelic profiles generated by *A. baumannii* core genome MLST (cgMLST) (6), *E. coli* cgMLST (7), and *S. aureus* cgMLST (8) (Figure S1). For MST construction, the option “pairwise ignore missing values” was applied. In the MSTs, genomes with an allelic distance of ≤ 9, ≤ 10, or ≤ 24 belonged to the same cluster according to the respective cgMLST scheme (*A. baumannii* cgMLST, *E. coli* cgMLST, *S. aureus* cgMLST).

## Construction of whole-genome sequencing reference data sets

The *S. aureus* and the *E. coli* whole-genome sequencing (WGS) reference data sets were constructed as follows: in brief, all *S. aureus* and *E. coli* complete genomes and chromosomes that were freely available on 22^nd^ of July and 31^st^ of August 2022, respectively, were downloaded from NCBI Genome database (<https://www.ncbi.nlm.nih.gov/genome/>). In summary, 853 complete *S. aureus* genomes and 192 *S. aureus* chromosomes as well as 2,368 complete *E. coli* genomes and 306 *E. coli* chromosomes were collected. Species level was confirmed by Mash Screen analysis (9). While all *S. aureus* genomes passed this test, 33 genomes formerly assigned to the species *E. coli* belonged to another species and therefore, were excluded from the data set. All remaining genomes were analyzed with either *S. aureus* cgMLST or *E. coli* cgMLST scheme, depending on the respective species. To construct WGS data sets of high quality, in both data sets genomes exhibiting less than 95 % of cgMLST genes were excluded, resulting in 993 *S. aureus* and 2,514 *E. coli* genomes per WGS data set. Based on the cgMLST profiles, for each data set a MST was generated to group similar genotypes, applying the option “pairwise ignore missing values”. In these MSTs, *S. aureus* and *E. coli* genomes with an allelic distance of ≤ 24 and ≤ 10, respectively, belonged to the same cluster. While in the MST comprising 993 *S. aureus* genomes 133 clusters were present, the MST including 2,514 *E. coli* genomes comprised 222 clusters. In both data sets, all individual, non-clustering genomes and the central genome of each cluster were kept, while the remaining 269 *S. aureus* and 590 *E. coli* genomes were removed, resulting in WGS reference data sets containing 724 and 1,924 heterogeneous *S. aureus* and *E. coli* genotypes, respectively. Subsequently, both WGS data sets were scaled down to 450 *S. aureus* and 472 *E. coli* genomes to ensure fast analysis time. In case of the *S. aureus* database, 274 genomes with an allelic distance of less than 100 to other genomes were excluded. Downscaling of the *E. coli* database was done in two steps. First, genomes that possessed a distance of less than 100 alleles to other genomes were excluded. Second, genomes with a distance of less than 500 alleles to other genomes were removed. All 1,045 *S. aureus* genomes and all 2,674 *E. coli* genomes used to construct the WGS reference data sets are listed in Table S2.

## Detection of cluster-specific single nucleotide polymorphisms

Single nucleotide polymorphism (SNP) analysis was performed as described recently (10). Briefly, the first two isolates of each cluster (*A. baumannii* OC050 and OC052, *E. coli* OC286 and OC287, *S. aureus* OC248 and OC249) as well as all genomes of the respective species-specific WGS reference data set were loaded in a comparison table. While for the *A. baumannii* and *S. aureus* comparison table, genes of the respective cgMLST and accessory schemes (*A. baumannii* cgMLST: 2,390 targets, *A. baumannii* accessory: 1,083 targets, *S. aureus* cgMLST: 1,861 targets, *S. aureus* accessory: 706 targets) were selected as table content (6, 8), the *E. coli* comparison table only contained data of the *E. coli* cgMLST scheme (2,513 targets) (7) as a public *E. coli* accessory scheme is missing. Based on the respective cgMLST and accessory profiles, MSTs were generated to group similar genotypes and to specify “Target-Genomes”, i. e. the two cluster isolates, and “Nontarget-Genomes”, i. e. sporadic isolates that do not belong to the cluster. While in the *S. aureus* and the *E. coli* MSTs only the two cluster isolates grouped together, in the *A. baumannii* MST strain OC051 of the *A. baumannii* WGS reference data set possessed the same genotype as the cluster strains and therefore, was added to the cluster. Via the function “Find Group Specific SNVs” (SNVs = single nucleotide variants), the search for cluster-specific SNPs was started. For this analysis, neither genomes nor columns with missing values were excluded. Missing values mean that either a cgMLST target or an accessory gene was not found at all in an input sequence, that the analysis for this target has failed, or that a new allele for this target has not yet been submitted (<https://www.ridom.de/u/Tutorial_for_SeqSphere+_Assembly_and_cgMLST_Analysis_Pipeline.html)>. The genomes of the cluster index isolates (i. e. strains *A. baumannii* OC050, *E. coli* OC286, *S. aureus* OC248) were selected as reference genome for extraction of cluster-specific SNPs. By definition, SNPs had to be identical in at least 100 % of the “Target-Genomes” and had to be different to the majority of “Target-Genomes” in at least 100 % of the “Nontarget-Genomes”. Furthermore, up to 300 neighboring bases on each side of the SNP should be found. Once SNP analysis was finished, SNPs that were insertions or deletions were excluded as they are not suitable for PCR detection. The remaining SNPs were exported to an Excel file that contained information about SNP localization, SNP base, alternative base at SNP position, influence on GC content and amino acid exchange, and the sequence containing the SNP.

## Alignment analysis with NCBI Nucleotide BLAST

NCBI Nucleotide BLAST analysis of SNP-containing sequences identified by SeqSphere^+^ was conducted as described recently (10). In more detail, a megablast analysis was performed to search for highly similar sequences between the SNP-containing sequences and genomes of the *A. calcoaceticus* – *A. baumannii* (ACB) complex (taxid: 909768), *E. coli* (taxid: 562), and *S. aureus* (taxid: 1280), respectively, stored in the Nucleotide Collection database. Uncultured or environmental sample sequences were excluded from analysis.

Per cluster, at least 20 SNP-containing sequences were investigated by Nucleotide BLAST analysis. If the BLAST analysis revealed that less than five out of 20 SNPs were cluster-specific, additional SNP-containing sequences were screened via BLAST.

## Extraction of genomic DNA

Genomic DNA analyzed by real-time PCR or WGS was extracted as described in Treffon *et al*. (10). In brief, the Monarch® Genomic DNA Purification Kit (New England Biolabs® GmbH, Frankfurt am Main, Germany) was used to isolate DNA of gram-negative and gram-positive bacteria, following the instructions of the manufacturer. For isolation of staphylococcal DNA, 5 µL of a 6 mg/mL lysostaphin solution were added to the lysis buffer, generating a working concentration of 0.15 mg/mL. DNA concentration was measured with NanoDrop^TM^ 2000c (Thermo Fisher Scientific, Wesel, Germany), Qubit 2.0 Fluorometer (Thermo Fisher Scientific), or DS-11 FX Fluorometer (Biozym, Hessisch Oldendorf, Germany) and DNA quality was checked by determination of A260/280 and A260/230 values. Samples with values < 1.4 were either treated according to the desalting/buffer exchange cleanup protocol from NEB or isolated new.

# Supplemental tables

Table S1 Bacterial strains analyzed in this study

| **Cluster** | **Species** | **Strain** | **Sequence type** | **NCBI accession(s)** |
| --- | --- | --- | --- | --- |
| - | *Acinetobacter baumannii* | OC045 | Unknown/  1327 | ERR628402 |
| - | *Acinetobacter baumannii* | OC046 | 10/447 | ERR628401 |
| - | *Acinetobacter baumannii* | OC047 | 10/585 | ERR628404 |
| - | *Acinetobacter baumannii* | OC048 | 10/1329 | ERR628405 |
| - | *Acinetobacter baumannii* | OC049 | 10/585 | ERR628410 |
| AB | *Acinetobacter baumannii* * | OC050 | 23/579 | ERR7281380 |
| AB | *Acinetobacter baumannii* | OC051 | 23/579 | ERR7281381 |
| AB | *Acinetobacter baumannii* | OC052 | 23/579 | ERR7281382 |
| SA | *Staphylococcus aureus* * | OC248 | 8 | ERR2685207 |
| SA | *Staphylococcus aureus* | OC249 | 8 | ERR2685208 |
| SA | *Staphylococcus aureus* | OC250 | 8 | ERR2685209, ERR2685210, ERR2685211 |
| SA | *Staphylococcus aureus* | OC251 | 8 | ERR2685212, ERR2685213, ERR2685214 |
| SA | *Staphylococcus aureus* | OC252 | 8 | ERR2685215 |
| SA | *Staphylococcus aureus* | OC253 | 8 | ERR2685216 |
| SA | *Staphylococcus aureus* | OC254 | 8 | ERR2685217 |
| - | *Staphylococcus aureus* | OC261 | 8 | ERR11894489 |
| - | *Staphylococcus aureus* | OC262 | 8 | ERR11894490 |
| - | *Staphylococcus aureus* | OC273 | 8 | ERR11894491 |
| - | *Staphylococcus aureus* | OC278 | 4804 | SAMEA115368948 |
| EC | *Escherichia coli* * | OC286 | 131 | ERR1195629 |
| EC | *Escherichia coli* | OC287 | 131 | ERR1195643 |
| EC | *Escherichia coli* | OC288 | 131 | ERR1195649 |
| EC | *Escherichia coli* | OC289 | 131 | ERR1195651 |
| EC | *Escherichia coli* | OC290 | 131 | ERR1195659 |
| EC | *Escherichia coli* | OC291 | 131 | ERR1195679 |
| - | *Escherichia coli* | OC293 | 131 | ERR11894483 |
| - | *Escherichia coli* | OC306 | 131 | ERR11894485, ERR11894486, ERR11894487 |
| - | *Escherichia coli* | OC307 | 131 | ERR11894484 |
| - | *Escherichia coli* | OC312 | 131 | ERR11894488 |
| - | *Acinetobacter baumannii* ^T^ | DSM 30007 | 52/931 | NZ_CP045110 |
| - | *Acinetobacter calcoaceticus* ^T^ | DSM 30006 |  | APQI01000000 |
| - | *Acinetobacter dijkshoorniae* ^T^ | LMG 29605 |  | LJPG01000000 |
| - | *Acinetobacter nosocomialis* ^T^ | DSM 102856 |  | APOP01000000 |
| - | *Acinetobacter pittii* ^T^ | DSM 25618 |  | APQP01000000 |
| - | *Acinetobacter seifertii* ^T^ | DSM 102854 |  | APOO01000000 |
| - | *Enterobacter cloacae* ^T^ | DSM 30054 |  | NC_014121 |
| - | *Enterococcus faecium* ^T^ | DSM 20477 |  | UFYJ01000000 |
| - | *Escherichia coli* ^T^ | DSM 30083 | 95 | NZ_CP033092 |
| - | *Klebsiella pneumoniae* ^T^ | DSM 30104 |  | JSZI01000000 |
| - | *Pseudomonas aeruginosa* ^T^ | DSM 50071 |  | NZ_CP012001 |
| - | *Staphylococcus aureus* ^T^ | DSM 20231 | 8 | NZ_CP035101 |
| - | *Staphylococcus epidermidis* ^T^ | DSM 20044 |  | NZ_CP035288 |

Sequence types (STs) for *A. baumannii*, *S. aureus*, and *E. coli* were determined via *A. baumannii* multilocus sequence typing (MLST) Pasteur (1) and Oxford (2), *S. aureus* MLST (4), and *E. coli* MLST Warwick (3), respectively, using SeqSphere^+^. *A. baumannii* Pasteur and Oxford STs are separated by a slash. Unidentified STs are indicated as “unknown”. Pasteur ST 10 is a single locus variant of Pasteur ST 23. *S. aureus* ST 4804 is a single locus variant of *S. aureus* ST 8. All strains except the type strains (^T^) were clinical isolates. While type strains were named according to their official culture collection number (prefix “DSM” or “LMG”), clinical isolates were labeled with an internal number (prefix “OC”). AB = *A. baumannii* cluster, EC = *E. coli* cluster, SA = *S. aureus* cluster, - = non-cluster strain, * = index isolate

**Table S3 Primer and probe sequences**

| **Cluster** | **Target** | **Sequences of primers and probes (5’ 🡪 3’)** | **Length of amplicon (nt)** |
| --- | --- | --- | --- |
| AB | Seq1AB | For: GGATCTTGCTTCAGTTGA  Rev: GCCTAGTTTATATGAGTCAGA  SNP probe: FAM-TTGTACTAAGC**T**CCGAATCACAT-BHQ1  Alternative probe: TTGTACTAAGC**C**CCGAATCACAT | 100 |
| AB | Seq7AB | For: ATGGGTTCATATATTCTAAAGC  Rev: AGCACCTTGATAATACATTTC  SNP probe: FAM-TGCCTAATATA**T**GCCCTGCTCCTA-BHQ1  Alternative probe: TGCCTAATATA**C**GCCCTGCTCCTA | 183 |
| AB | Seq12AB | For: CACCATACCAATAATTAGGC  Rev: GGGACCGTATGTTACAAA  SNP probe: FAM-TCCACGGCC**C**TATGAAGCTA-BHQ1  Alternative probe: TCCACGGCC**A**TATGAAGCTA | 200 |
| EC | Seq15EC | For: CGACAACTGTTACAGGAA  Rev: GTCAGGTGGTTGATATGG  SNP probe: FAM-ATCCAGGAAAA**T**AGACCACATCAGC-BHQ1  Alternative probe: ATCCAGGAAAA**C**AGACCACATCAGC | 200 |
| EC | Seq25EC | For: GTCAGCAGAATATTAATAATGAAC  Rev: CTGGAGAATCGTCATCAC  SNP probe: FAM-ATCATTCTG**T**CGCCGCTCGG-BHQ1  Alternative probe: ATCATTCTG**C**CGCCGCTCGG | 93 |
| EC | Seq31EC | For: GGCAAGTTGATTGGTGAC  Rev: AGCCTTTCTTACGGTCAA  SNP probe: FAM-CGACGCTGGCC**T**CAACGGTG-BHQ1  Alternative probe: CGACGCTGGCC**C**CAACGGTG | 112 |
| SA | Seq1SA | For: ATGCGAGATGTTATTAACAA  Rev: CGTTCAATGGCTTCTTTA  SNP probe: FAM-TTGGCACCAC**C**TACAGCTAAA-BHQ1  Alternative probe: TTGGCACCAC**T**TACAGCTAAA | 89 |
| SA | Seq4SA | For: GACGGCTATATGGTTGTTA  Rev: CGCTAAGGCTTCAAAGTTA  SNP probe: FAM-ATGTTCAATC**G**CATCACTCGCA-BHQ1  Alternative probe: ATGTTCAATC**A**CATCACTCGCA | 174 |
| SA | Seq5SA | For: CGGTTGAAGAAGTTTTAATC  Rev: CGATGTGAATCTTCTAAGAA  SNP probe: FAM-ACATCGAACCA**A**TACAAGCACCT-BHQ1  Alternative probe: ACATCGAACCA**T**TACAAGCACCT | 187 |
| - | 16S rDNA | For: CAGACTCCTACGGGAG  Rev: GACTACCAGGGTATCTAATC  Probe: Cy5-GTGCCAGCAGCCGCGGTAATAC-BHQ2 | 472 |

Single nucleotide polymorphism and alternative nucleotides are highlighted in blue in the respective probe sequence. AB = *A. baumannii* cluster, BHQ = Black Hole Quencher, Cy5 = cyanine-5, EC = *E. coli* cluster, FAM = fluorophore 6-carboxyfluorescein, For = forward primer, nt = nucleotides, Rev = reverse primer, SA = *S. aureus* cluster

**Table S4 Location of cluster-specific SNPs selected for real-time PCR**

| **Cluster** | **SNP ID** | **Locus tag** | **Gene** | **Product** | **Absolute SNP position in ref. genome** | **SNP in cluster isolates** | **Alternative nucleotide in ref. genome** | **GC content change** | **Mutation type** |
| --- | --- | --- | --- | --- | --- | --- | --- | --- | --- |
| AB | Seq1AB | ACICU_RS01355 | N/D | Peptidase S41 | 278510 | T | C | Yes | Non-synonymous |
|  | Seq7AB | ACICU_RS05660 | N/D | Microcin C ABC transporter permease YejB | 1189895 | A | G | Yes | Non-synonymous |
|  | Seq12AB | ACICU_RS14210 | N/D | AI-2E family transporter | 3004337 | G | T | Yes | Synonymous |
| EC | Seq15EC | b1423 | *ydcJ* | Putative metalloenzyme | 1495934 | A | G | Yes | Non-synonymous |
|  | Seq25EC | b2666 | *yqaE* | CyaR sRNA-regulated protein | 2796992 | A | G | Yes | Non-synonymous |
|  | Seq31EC | b4227 | *ytfQ* | Galactofuranose ABC transporter periplasmic binding protein | 4450440 | A | G | Yes | Non-synonymous |
| SA | Seq1SA | SACOL0130 | N/D | 5' nucleotidase | 146938 | G | A | Yes | Non-synonymous |
|  | Seq4SA | SACOL0636 | *mvk* | Mevalonate kinase | 668811 | C | T | Yes | Non-synonymous |
|  | Seq5SA | SACOL0638 | N/D | Phosphomevalonate kinase | 670710 | A | T | No | Non-synonymous |

Information were extracted with SeqSphere^+^. All single nucleotide polymorphisms (SNPs) are located in the core genome. As reference genomes for the *A. baumannii* cluster, the *S. aureus* cluster, and the *E. coli* cluster the sequences of *A. baumannii* ACICU (accession: NC_010611), *S. aureus* subsp. *aureus* COL (accession: NC_002951), and *E. coli* str. K-12 substr. MG1655 (accession: NC_000913) were applied, respectively. AB = *A. baumannii* cluster, EC = *E. coli* cluster, ID = identifier, N/D = not defined, ref. = reference, SA = *S. aureus* cluster

Table S5 **Probe-based real-time PCR detection of cluster isolates in 96-well plates**

| **Cluster** | **Species** | **Strain** | **DNA copies** | **Target** | **Ct value** |
| --- | --- | --- | --- | --- | --- |
| AB | *A. baumannii* | OC050 | 100 | Seq1AB | 30.22 |
| AB | *A. baumannii* | OC052 | 100 | Seq1AB | 33.10 |
| AB | *A. baumannii* | OC050 | 10,000 | Seq1AB | 23.93 |
| AB | *A. baumannii* | OC052 | 10,000 | Seq1AB | 23.83 |
| - | *A. baumannii* | OC045 | 10,000 | Seq1AB | N/D |
| - | *A. baumannii* | OC046 | 10,000 | Seq1AB | N/D |
| - | *A. baumannii* | OC047 | 10,000 | Seq1AB | N/D |
| - | *A. baumannii* | OC049 | 10,000 | Seq1AB | N/D |
| - | *A. baumannii* | DSM 30007 | 10,000 | Seq1AB | N/D |
| - | - | H_2_O | - | Seq1AB | N/D |
| AB | *A. baumannii* | OC050 | 100 | Seq7AB | 33.64 |
| AB | *A. baumannii* | OC052 | 100 | Seq7AB | 30.08 |
| AB | *A. baumannii* | OC050 | 10,000 | Seq7AB | 23.32 |
| AB | *A. baumannii* | OC052 | 10,000 | Seq7AB | 23.79 |
| - | *A. baumannii* | OC045 | 10,000 | Seq7AB | N/D |
| - | *A. baumannii* | OC046 | 10,000 | Seq7AB | N/D |
| - | *A. baumannii* | OC047 | 10,000 | Seq7AB | N/D |
| - | *A. baumannii* | OC049 | 10,000 | Seq7AB | N/D |
| - | *A. baumannii* | DSM 30007 | 10,000 | Seq7AB | N/D |
| - | - | H_2_O | - | Seq7AB | N/D |
| AB | *A. baumannii* | OC050 | 100 | Seq12AB | 31.45 |
| AB | *A. baumannii* | OC052 | 100 | Seq12AB | 32.16 |
| AB | *A. baumannii* | OC050 | 10,000 | Seq12AB | 24.06 |
| AB | *A. baumannii* | OC052 | 10,000 | Seq12AB | 23.97 |
| - | *A. baumannii* | OC045 | 10,000 | Seq12AB | N/D |
| - | *A. baumannii* | OC046 | 10,000 | Seq12AB | N/D |
| - | *A. baumannii* | OC047 | 10,000 | Seq12AB | N/D |
| - | *A. baumannii* | OC049 | 10,000 | Seq12AB | N/D |
| - | *A. baumannii* | DSM 30007 | 10,000 | Seq12AB | N/D |
| - | - | H_2_O | - | Seq12AB | N/D |
| EC | *E. coli* | OC286 | 100 | Seq15EC | 30.60 |
| EC | *E. coli* | OC287 | 100 | Seq15EC | 30.82 |
| EC | *E. coli* | OC286 | 10,000 | Seq15EC | 24.14 |
| EC | *E. coli* | OC287 | 10,000 | Seq15EC | 24.21 |
| - | *E. coli* | OC293 | 10,000 | Seq15EC | 26.95 |
| - | *E. coli* | OC306 | 10,000 | Seq15EC | 21.38 |
| - | *E. coli* | OC307 | 10,000 | Seq15EC | 21.85 |
| - | *E. coli* | OC312 | 10,000 | Seq15EC | 24.84 |
| - | *E. coli* | DSM 30083 | 10,000 | Seq15EC | N/D |
| - | - | H_2_O | - | Seq15EC | N/D |
| EC | *E. coli* | OC286 | 100 | Seq25EC | 30.83 |
| EC | *E. coli* | OC287 | 100 | Seq25EC | 31.09 |
| EC | *E. coli* | OC286 | 10,000 | Seq25EC | 23.84 |
| EC | *E. coli* | OC287 | 10,000 | Seq25EC | 23.90 |
| - | *E. coli* | OC293 | 10,000 | Seq25EC | 21.26 |
| - | *E. coli* | OC306 | 10,000 | Seq25EC | 24.53 |
| - | *E. coli* | OC307 | 10,000 | Seq25EC | 26.59 |
| - | *E. coli* | OC312 | 10,000 | Seq25EC | 22.62 |
| - | *E. coli* | DSM 30083 | 10,000 | Seq25EC | 26.50 |
| - | - | H_2_O | - | Seq25EC | N/D |
| EC | *E. coli* | OC286 | 100 | Seq31EC | 30.85 |
| EC | *E. coli* | OC287 | 100 | Seq31EC | 31.90 |
| EC | *E. coli* | OC286 | 10,000 | Seq31EC | 23.90 |
| EC | *E. coli* | OC287 | 10,000 | Seq31EC | 23.71 |
| - | *E. coli* | OC293 | 10,000 | Seq31EC | 23.59 |
| - | *E. coli* | OC306 | 10,000 | Seq31EC | 22.94 |
| - | *E. coli* | OC307 | 10,000 | Seq31EC | 22.83 |
| - | *E. coli* | OC312 | 10,000 | Seq31EC | 22.51 |
| - | *E. coli* | DSM 30083 | 10,000 | Seq31EC | 22.83 |
| - | - | H_2_O | - | Seq31EC | N/D |
| SA | *S. aureus* | OC248 | 100 | Seq1SA | 30.95 |
| SA | *S. aureus* | OC249 | 100 | Seq1SA | 30.30 |
| SA | *S. aureus* | OC248 | 10,000 | Seq1SA | 22.51 |
| SA | *S. aureus* | OC249 | 10,000 | Seq1SA | 22.80 |
| - | *S. aureus* | OC261 | 10,000 | Seq1SA | N/D |
| - | *S. aureus* | OC262 | 10,000 | Seq1SA | N/D |
| - | *S. aureus* | OC273 | 10,000 | Seq1SA | N/D |
| - | *S. aureus* | OC278 | 10,000 | Seq1SA | N/D |
| - | *S. aureus* | DSM 20231 | 10,000 | Seq1SA | N/D |
| - | - | H_2_O | - | Seq1SA | N/D |
| SA | *S. aureus* | OC248 | 100 | Seq4SA | 31.12 |
| SA | *S. aureus* | OC249 | 100 | Seq4SA | 31.58 |
| SA | *S. aureus* | OC248 | 10,000 | Seq4SA | 23.20 |
| SA | *S. aureus* | OC249 | 10,000 | Seq4SA | 23.12 |
| - | *S. aureus* | OC261 | 10,000 | Seq4SA | N/D |
| - | *S. aureus* | OC262 | 10,000 | Seq4SA | N/D |
| - | *S. aureus* | OC273 | 10,000 | Seq4SA | N/D |
| - | *S. aureus* | OC278 | 10,000 | Seq4SA | N/D |
| - | *S. aureus* | DSM 20231 | 10,000 | Seq4SA | 20.88 |
| - | - | H_2_O | - | Seq4SA | N/D |
| SA | *S. aureus* | OC248 | 100 | Seq5SA | 37.18 |
| SA | *S. aureus* | OC249 | 100 | Seq5SA | 31.65 |
| SA | *S. aureus* | OC248 | 10,000 | Seq5SA | 24.91 |
| SA | *S. aureus* | OC249 | 10,000 | Seq5SA | 24.74 |
| - | *S. aureus* | OC261 | 10,000 | Seq5SA | N/D |
| - | *S. aureus* | OC262 | 10,000 | Seq5SA | N/D |
| - | *S. aureus* | OC273 | 10,000 | Seq5SA | N/D |
| - | *S. aureus* | OC278 | 10,000 | Seq5SA | N/D |
| - | *S. aureus* | DSM 20231 | 10,000 | Seq5SA | N/D |
| - | - | H_2_O | - | Seq5SA | N/D |

Ct values of real-time PCR curves depicted in Figure 2 are shown. AB = *A. baumannii* cluster, EC = *E. coli* cluster, H_2_O = non-target control, N/D = no signal detection, SA = *S. aureus* cluster, - = Non-cluster strain

# Supplemental figures


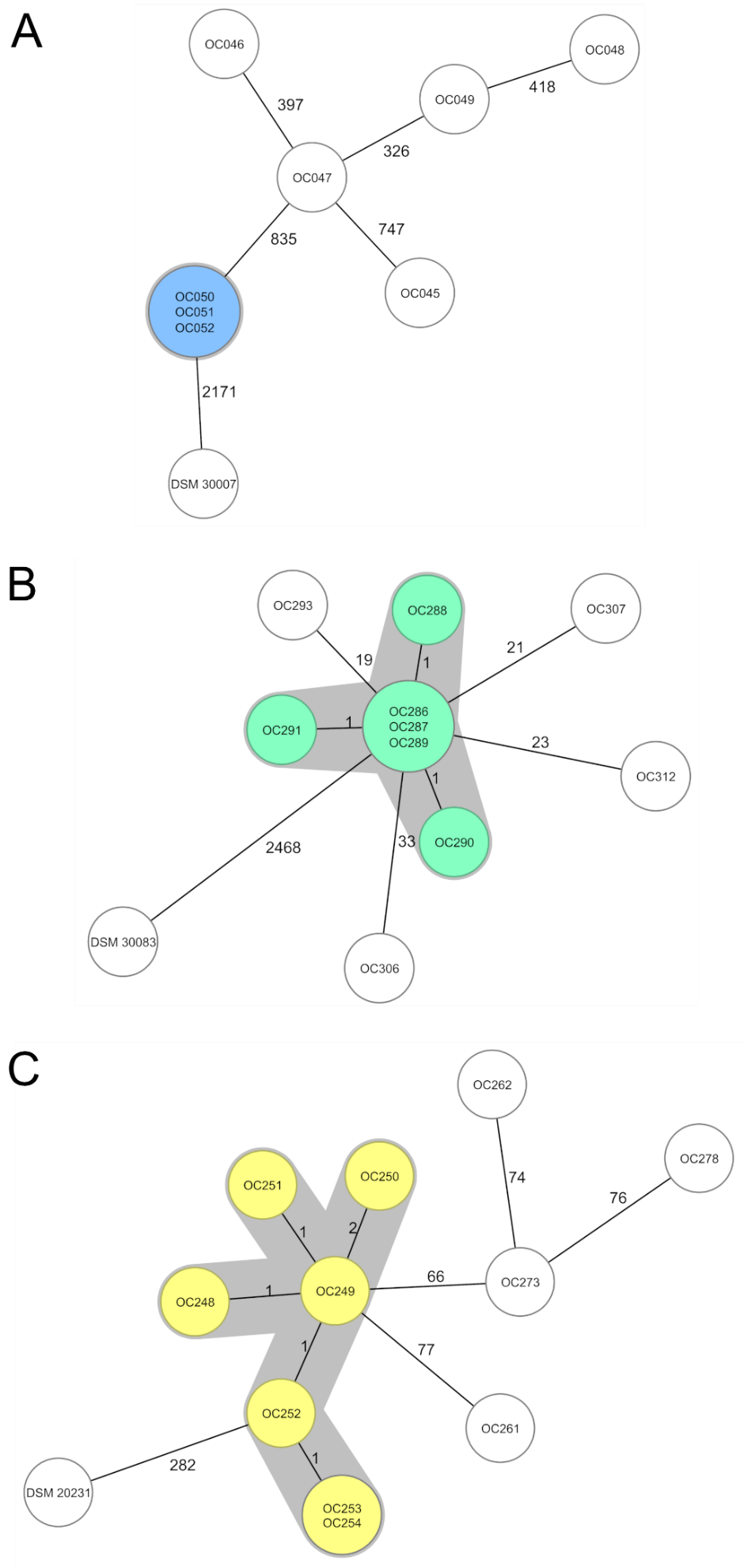


**Figure S1 Minimum spanning tree (MST) of bacterial strains used for validation of real-time PCR primers and probes. A:** MST of nine *A. baumannii* strains. **B:** MST of 11 *E. coli* strains. **C:** MST of 12 *S. aureus* strains. The MSTs were constructed based on the allelic profile generated by *A. baumannii* core genome multilocus sequence typing (cgMLST), *E. coli* cgMLST, and *S. aureus* cgMLST, respectively, using SeqSphere^+^. For MST construction, the option “pairwise ignore missing values” was applied. Nodes represent individual genotypes and are named by the strains. The size of the nodes is proportional to the number of strains of the same genotype. The numbers of differing alleles between the genotypes are indicated at the connection lines between the nodes. Isolates with an allelic distance ≤ 9, ≤ 10, or ≤ 24 belong to the *A. baumannii* cluster, the *E. coli* cluster, or the *S. aureus* cluster, respectively. Cluster isolates are shaded in grey and have the same node color.


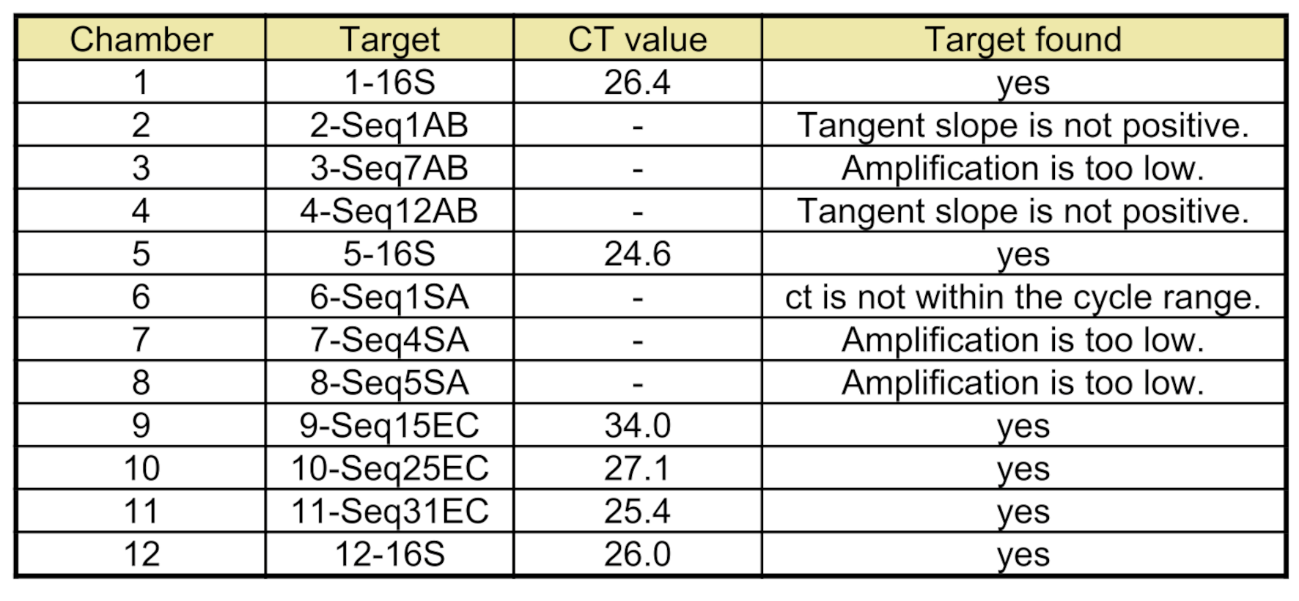


**Figure S2 Cutout of the report produced by the TSO analyzer software after analysis of *E. coli* cluster isolate OC288.** The report provides data regarding PCR chamber, PCR target, Ct value measured for this target, and a notification if the target was found. In case the target was not detected, the report notifies why the detection failed.


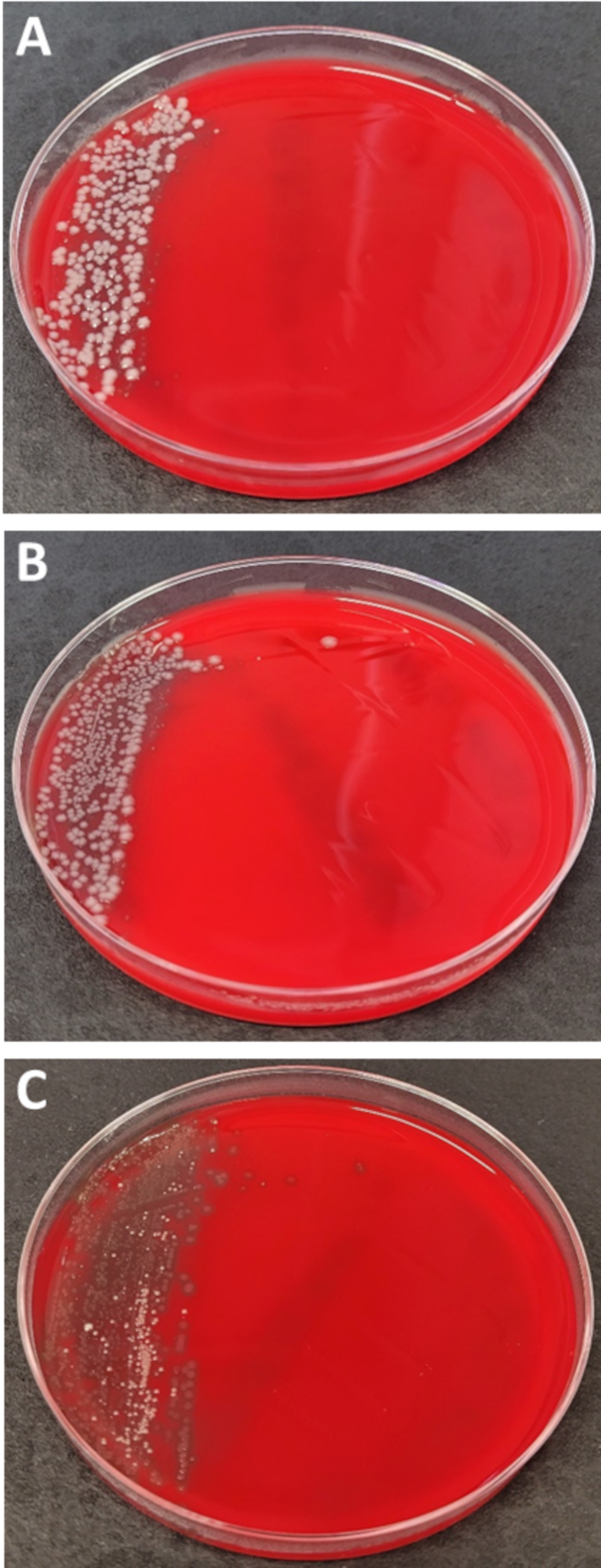


**Figure S3 Eluate of swab samples streaked on blood agar.** Moisturized swabs rubbed over the oral mucosa of healthy volunteers were either spotted with 10^5^ CFU of cluster isolates *A. baumannii* OC050 (A) or *E. coli* OC286 (B) or were left unspotted (C). Swabs were eluted in 3 mL of 0.9 % NaCl solution, which was subjected to real-time analysis with the microfluidic system. Before, 10 µL of each eluate were plated on blood agar and incubated overnight at 37 °C to control bacterial growth.

# Reference

1. Diancourt L, Passet V, Nemec A, Dijkshoorn L, Brisse S. 2010. The population structure of *Acinetobacter baumannii*: expanding multiresistant clones from an ancestral susceptible genetic pool. PLoS One 5:e10034.

2. Bartual SG, Seifert H, Hippler C, Luzon MAD, Wisplinghoff H, Rodríguez-Valera F. 2005. Development of a multilocus sequence typing scheme for characterization of clinical isolates of *Acinetobacter baumannii*. J Clin Microbiol 43:4382–90.

3. Wirth T, Falush D, Lan R, Colles F, Mensa P, Wieler LH, Karch H, Reeves PR, Maiden MCJ, Ochman H, Achtman M. 2006. Sex and virulence in *Escherichia coli*: an evolutionary perspective. Mol Microbiol 60:1136–51.

4. Enright MC, Day NPJ, Davies CE, Peacock SJ, Spratt BG. 2000. Multilocus sequence typing for characterization of methicillin-resistant and methicillin-susceptible clones of *Staphylococcus aureus*. J Clin Microbiol 38:1008–15.

5. Jünemann S, Sedlazeck FJ, Prior K, Albersmeier A, John U, Kalinowski J, Mellmann A, Goesmann A, Von Haeseler A, Stoye J, Harmsen D. 2013. Updating benchtop sequencing performance comparison. Nat Biotechnol 31:294–6.

6. Higgins PG, Prior K, Harmsen D, Seifert H. 2017. Development and evaluation of a core genome multilocus typing scheme for whole-genome sequence-based typing of *Acinetobacter baumannii*. PLoS One 12:e0179228.

7. Zhou Z, Alikhan NF, Mohamed K, Fan Y, Achtman M. 2020. The EnteroBase user’s guide, with case studies on *Salmonella* transmissions, *Yersinia pestis* phylogeny, and *Escherichia* core genomic diversity. Genome Res 30:138–52.

8. Leopold SR, Goering R V., Witten A, Harmsen D, Mellmann A. 2014. Bacterial whole-genome sequencing revisited: portable, scalable, and standardized analysis for typing and detection of virulence and antibiotic resistance genes. J Clin Microbiol 52:2365–70.

9. Ondov BD, Starrett GJ, Sappington A, Kostic A, Koren S, Buck CB, Phillippy AM. 2019. Mash Screen: high-throughput sequence containment estimation for genome discovery. Genome Biol 20:232.

10. Treffon J, Heppner B, Eismann J, Bothe J, Omengo B, Mellmann A. 2022. Single nucleotide polymorphism-based real-time PCR screening assay for rapid tracking of bacterial infection clusters to complement whole-genome sequencing efforts during outbreak investigations. Microbiol Spectr 10:e0303622.
